# Mouse models of colorectal cancer as preclinical models

**DOI:** 10.1002/bies.201500032

**Published:** 2015-06-26

**Authors:** Rebecca E. McIntyre, Simon J.A. Buczacki, Mark J. Arends, David J. Adams

**Affiliations:** ^1^ Experimental Cancer GeneticsWellcome Trust Sanger InstituteHinxtonCambridgeUK; ^2^ Li Ka Shing CentreCancer Research UK Cambridge InstituteCambridgeUK; ^3^ Edinburgh Cancer Research UK CentreUniversity of EdinburghEdinburghUK

**Keywords:** colorectal cancer, disease biomarker, mouse model, ome and omics, target identification, target validation

## Abstract

In this review, we discuss the application of mouse models to the identification and pre‐clinical validation of novel therapeutic targets in colorectal cancer, and to the search for early disease biomarkers. Large‐scale genomic, transcriptomic and epigenomic profiling of colorectal carcinomas has led to the identification of many candidate genes whose direct contribution to tumourigenesis is yet to be defined; we discuss the utility of cross‐species comparative ‘omics‐based approaches to this problem. We highlight recent progress in modelling late‐stage disease using mice, and discuss ways in which mouse models could better recapitulate the complexity of human cancers to tackle the problem of therapeutic resistance and recurrence after surgical resection.

**Abbreviations**
CRC: colorectal cancer
EGF: epidermal growth factor
GEMM: genetically engineered mouse models
HNPCC: hereditary non‐polyposis CRC
SNPs: single‐nucleotide polymorphisms
Tfh: T follicular helper
TGFβ: transforming growth factor β

## Introduction

Colorectal cancer (CRC) accounts for 700,000 deaths and 1.4 million newly diagnosed cases globally per annum, making it the number one cause of non‐smoking related cancer deaths 1. Most CRCs arise in the epithelium, a process driven by genetic and/or epigenetic alterations that result in the formation of premalignant lesions called adenomas (see Table 1 for list of genes most frequently mutated in CRCs). The transition from normal epithelium to benign adenoma is initially driven by alterations that hyperactivate the Wnt pathway (>95% CRCs), and this predominantly occurs through inactivation of the *APC* gene (>80% CRCs) 2, 3, 4. APC is a negative regulator of the Wnt pathway and hyperactivation of the Wnt pathway is critical to both the initiation and maintenance of the vast majority of CRCs, although pathway activation is finely tuned 2, 5, 6. For example, alteration of many genes that are known to regulate Wnt signalling, such as *AXIN2* (Axis inhibition protein 2) and *AMER1* (APC membrane recruitment 1) are found in tumours that also harbour *APC* mutations 2, 7. The Wnt pathway is also critical for the maintenance of the intestinal epithelium, which undergoes complete renewal every 4–5 days in humans 8; therefore, it has not been possible to target this pathway in cancers without disrupting intestinal regeneration 9.

**Table 1:** Selection of some of the most frequently mutated genes in colorectal cancer

Human/mouse gene symbol	Frequency (%)	Full gene name	Role in development of CRC
*APC/Apc*	>80	Adenomatous Polyposis Coli	• Inactivation of APC is the initiating event in the majority of colorectal cancers 100
			• APC is a negative regulator of the Wnt pathway
			• Hyperactivation of the Wnt pathway initiates development of CRC by stabilising the β‐catenin transcription factor, increasing expression of transcription factors such as c‐Myc and c‐Fos, which regulate expression of cell cycle genes
*TP53/Trp53*	∼65	Tumor protein p53	• Loss of TP53 is associated with disease progression 101
			• TP53 regulates expression of genes that induce cell cycle arrest, apoptosis, senescence, DNA repair, or changes in metabolism
*KRAS/Kras*	∼45	Kirsten rat sarcoma viral oncogene homolog	• Mutation of KRAS (>75% of mutations are at codon 12) and BRAF cause activation of the Ras‐MAPK pathway and mutations in these genes are mutually exclusive 2
			• Activation of Ras‐Mapk pathway is associated with disease progression and poor prognosis 102
*BRAF/Braf*	∼8	v‐raf murine sarcoma viral oncogene homolog B	• Activation of the Ras‐MAPK pathway leads to activation of transcription factors such as c‐Myc, and c‐Fos, which in turn regulate expression of cell cycle genes
*FBXW7/Fbxw7*	∼20	F‐Box And WD Repeat Domain Containing 7	• E3 ubiquitin ligase that targets cyclin E and c‐Myc (cell cycle regulators) for ubiquitin‐mediated degradation 103
			• Low expression of FBXW7 is associated with poor prognosis 104
*TGFBR2/Tgfbr2*	∼12	Transforming growth factor, beta receptor 2	• Alterations in TGFβ pathway e.g. mutation of *TGFBR2*, *SMAD2*, *SMAD3* and *SMAD4*, are associated with disease progression 93, 105
*SMAD2/Smad2*	∼7	SMAD family member 2, 3, 4	• The SMAD family are intracellular signal transducers that are activated by TGFβ receptors and act as transcriptional modulators.
*SMAD3/Smad3*	∼5		
*SMAD4/Smad4*	∼12		
*PTEN/Pten*	∼10	Phosphatase and tensin homolog	• PTEN dephosphorylates phosphoinositide substrates and negatively regulates intracellular levels of phosphatidylinositol‐3,4,5‐trisphosphate (PIP3)
			• Negatively regulates AKT/PKB signaling pathway
*CDK8/Cdk8*	∼12	Cyclin dependent kinase 8	• CDK8 regulates the cell cycle by acting as a co‐activator for transcription of nearly all RNA Polymerase II‐dependent genes

Activation of the Ras‐MAPK (Kirsten rat sarcoma viral oncogene homolog and mitogen activated protein kinase) pathway, usually via point mutations that constitutively activate KRAS or BRAF (v‐raf murine sarcoma viral oncogene homolog B), and inactivation of the TGFβ (transforming growth factor β) pathway through inactivation of *SMAD* gene family members or TGFβ receptors, promote the development of advanced adenomas or invasive adenocarcinomas 3. Over time, a small proportion of advanced adenomas acquire further molecular abnormalities that transform them to invasive and then metastatic carcinomas. Inactivation of *TP53* (Tumour protein p53) or *IGF2R* (Insulin‐like growth factor 2 receptor) occurs with greater frequency in established carcinomas that invade submucosal layers than in adenomas 3, 10. However the molecular alterations that support metastases are poorly understood; at the genomic level at least, it would appear that there is high concordance between primary CRCs and their matched metastatic lesions, which suggests that mechanisms other than gene mutation may be responsible for progression to metastatic disease—for example epigenetic or post‐translational modifications 10, 11, 12.

Endoscopic or surgical resection is routinely used for patients with early premalignant adenomas, as well as for the treatment of most early stage carcinomas and selected patients with late stage or advanced metastatic disease, for whom chemotherapy (or radiotherapy/chemoradiotherapy for rectal cancers) is also a key treatment modality 13. Liver metastases of colorectal carcinomas occur in about 50% of patients, either at the time of diagnosis or at recurrence, and this is a major cause of CRC‐related deaths 13. Long‐term survival of CRC patients is correlated with disease stage at diagnosis, and the 5‐year survival rate for patients with metastatic CRC is less than 10% 14.

Aside from our lack of understanding of the molecular events that drive metastases, there are several other plausible explanations for our limited success in treating CRC. Firstly, there are currently few biomarkers that are predictive of early disease, the likelihood of favourable treatment response, recurrence or the development of metastatic disease 10. Secondly, therapeutic resistance, be it acquired or intrinsic, to several licensed colorectal cancer therapies is a major problem 15. For example, overexpression of the epidermal growth factor (EGF) receptor is associated with increased metastatic potential and poor prognosis in CRC. In these subgroups of cancer patients, monoclonal antibodies to the EGF receptor e.g. Cetuximab and Panitumumab, block EGF binding and result in tumour regression 16, 17. Resistance to therapy eventually develops through a variety of mechanisms including point mutations in the EGF receptor that inhibit Cetuximab binding but not EGF, or activating mutations in *KRAS*, an intracellular signalling pathway that converges with the EGF receptor pathway. Finally, the drug discovery pipeline attrition rate for oncology investigational compounds is extremely high. Only 5% of compounds that show promise in pre‐clinical studies are eventually approved for clinical use as the majority fail in Phase I/II trials 18. One of the reasons for this may be the extensive use of CRC cell lines and xenograft models derived from them in pre‐clinical anti‐cancer drug testing (reviewed by 18, 19). Cell culture does not model the interaction of primary tumour cells with surrounding cells (the tumour microenvironment or stroma), the process of organ colonisation by metastatic cancer cells (the ‘seed and soil’ hypothesis), and the recruitment and activation of the adaptive and innate immune systems during tumourigenesis, which are known to exert selective pressures on cancer cells 20, 21, 22. Several cell culture systems have been developed to make allowances for reciprocal cell communication; examples include organoid cultures, the co‐culture of tumour epithelial cells and stromal cells or three‐dimensional cultures 23, 24, 25. While these systems provide important insights into disease biology, they are limited in their ability to model the dynamic interplay between tumour and host. In this review, we summarise the strengths and weaknesses of xenograft models derived from CRC cell lines and primary patient derived material, and genetically modified mouse models of colorectal cancer. We discuss several novel applications of these models in cancer gene validation, biomarker discovery and target validation, and discuss their future roles in cross‐species comparative ‘omics’ approaches to understanding metastasis and therapeutic resistance.

## Cell line xenografts for reductionist target validation

**Figure 1:**
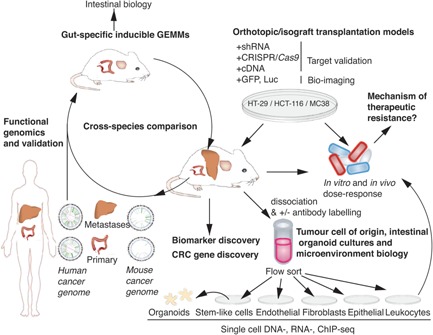
Mouse models of advanced colorectal cancer (CRC) as research and preclinical tools. The development of genetically engineered mouse models (GEMMs) carrying gene mutations that closely match those found by deep‐sequencing of human colorectal carcinomas provide information on the homeostatic and tumourigenic role of a gene in the intestine. Orthotopic transplantation of select colorectal carcinoma cell lines to the caecum of immune‐deficient mice results in primary tumour growth and metastases to lymph nodes or liver. Prior to transplantation, colorectal carcinoma cell lines can be genetically manipulated to overexpress (cDNA vector) or inhibit (shRNA or CRISPR‐*Cas9* vector) a gene of interest to rapidly assess its biological role or to validate potential therapeutic targets. Introducing a reporter gene, such as green fluorescent protein (GFP), to cells prior to transplantation allows in vivo monitoring of primary tumour or metastases growth, which is extremely useful when assessing responses to pharmacological agents or inducible genetic manipulation. Single‐cell suspensions from carcinomas can be flow‐sorted using cell‐specific markers to identify cells of interest, for example stem‐like cells for growth of organoids, or to gain further insight into the tumour cells of origin and their interactions with the microenvironment. Cultured cells can be utilised for drug sensitivity screens. Mouse models of advanced CRC can be used to screen for cancer driver genes and disease biomarkers.

Xenografting involves the injection of cells or the surgical transplantation of primary tissue into immune‐deficient mice such as the nude mouse, which is athymic and does not produce T‐lymphocytes, or the severe combined immune‐deficient (SCID) mouse, which has altered adaptive immunity but normal innate immunity, to prevent rejection. It was thought that the large number of commercial colorectal cell lines available would represent the inter‐tumour heterogeneity of the human disease, but in reality, few cell lines lead to reliable primary tumour growth, and fewer still to naturally metastatic CRC 26. Success rates appear to be highest when using the colorectal carcinoma cell lines HCT‐116 or HT‐29, but it is not yet clear why this is. Xenograft models are useful for rapid, reductionist, target validation studies, including validation of the many candidate cancer genes identified by The Cancer Genome Atlas (TCGA), however the results must be interpreted with caution (Table 2, Fig. 1 27, 28). For example, the environment of subcutaneous tumours is very different from that of autochthonous colorectal carcinomas (i.e. tumours that originate in the location where they are found) and liver metastases; and the extent to which the species mismatch in tumour (human) and stromal (mouse) cells influence tumour growth in vivo is uncertain. CRC stroma is composed of macrophages that secrete matrix‐degrading factors such as metalloproteinases, myofibroblasts that secrete Wnt ligands, T‐cells that secrete proinflammatory cytokines, and endothelial cells and bone‐marrow derived cells, all of which contribute greatly to the growth and progression of this tumour type 20, 22, 29.

**Table 2:** Transplantation models of colorectal cancer (CRC)

Transplantation model	Strengths	Weaknesses
Subcutaneous CRC cell line xenograft: injection of cells e.g. HCT116 and HT29 to immune deficient mouse	• Low cost	• Representative of advanced disease
	• Rapid tumour growth (2 weeks)	• Have undergone significant clonal selection 73
	• Well characterized cell‐lines: gene mutation status, transcriptome and drug response data available 73, 106, 107	• Microenvironmental differences (cells derived from colorectum injected under skin)
	• Easy to genetically manipulate prior to transplantation e.g. with inducible shRNA or by CRISPR/Cas9 108	• Species mismatch in tumour (human) and stromal (mouse) cell may limit cross‐talk
	• Model accessible to the majority of research labs	• Immune deficient host
		• Rarely metastasise
Orthotopic xenografts of CRC cell lines: injection of cells into intestinal serosa of immune deficient mouse	• As above	• As above (except for ‘rarely metastasise’)
	• More natural microenvironment for CRC cells	• May require surgery to implant cells
	• Some metastasise to liver e.g. from HCT116 or HT29	
Patient‐derived xenografts (PDXs): Suturing of 1–2 mm fragments of fresh surgical specimens of CRC, to intestine of immune deficient mouse	• Reproducible liver metastasis	• Not readily scalable
	• Avoid selection of dominant clones during long‐term cell culture	• Host (mouse) stromal cells replace human stromal cells within a few weeks (species mismatch)
	• Temporary preservation of species‐specific tumour‐stromal cell cross talk	• Immune deficient host
	• More natural microenvironment for CRC cells	• Limited by availability of surgical specimens
		• Expensive (labour intensive and time consuming)
Syngraft/Isograft: Suturing 1–2 mm mouse tumour fragments or mouse tumour cell lines e.g. MC38 cells to a genetically identical inbred, immune competent mouse	• No species mismatch between tumour and stromal cells	• Expensive (labour intensive and time consuming)
	• Host has intact immune system that enables testing of immunomodulatory anti‐cancer agents	• The model is not human

## Orthotopic xenografts of CRC cell lines improve tumour‐stromal cell cross‐talk and the reproducibility of liver metastases

Microenvironmental differences likely explain why the morphology of subcutaneous CRC cell line xenografts, especially the stromal elements, are often markedly different from the human disease and may explain why they have a low metastatic potential 30. In an attempt to improve the utility of xenograft models, several investigators have systematically tested different variables and found that CRC cell line, site of injection, mouse age and the genetic background of the mouse all markedly affect the frequency of lymph‐node or liver metastases 27, 31, 32. Orthotopic xenografts involve the injection of differentiated cells to a more natural microenvironment i.e. the serosa of the intestine for CRC cell lines, and appears to result in more reproducible liver metastasis 31, 33. In addition, orthotopic and subcutaneous xenograft models show differing sensitivities to chemotherapeutic agents 30, 31, suggesting that the microenvironment is important for both disease progression and therapeutic response. Recent refinements to the caecal pouch xenograft method include the non‐surgical trans‐anal injection of cells into the distal rectum or the microinjection of cell lines to the caecum and engineering cells to express β‐human chorionic gonadotropin to support growth 31, 34.

## Patient‐derived orthotopic xenografts temporarily preserve tumour‐stromal cell cross‐talk and improves reproducibility of metastasis

Patient derived xenografts (or ‘PDXs’) avoid the natural selection of dominant clones and epigenetic and genetic alterations that occur during long‐term cell culture as well as temporarily preserving the original, species‐specific, tumour‐stromal cell interactions. The suturing of small fragments of fresh surgical specimens of CRC liver metastases to the caecum of immune‐deficient mice has been reproducible in generating models of metastatic CRC 35, 36. It was thought that PDXs could be used to test therapies and development of resistance ahead of patient treatment to help personalize cancer therapy, however the approach requires subculture or serial xenografting which currently takes too long 37. A number of studies have also now reported that host cells replace the human stroma and vasculature of patient derived xenografts, and in the case of CRCs, this appears to occur relatively rapidly (3 weeks vs. 9 weeks for mesothelioma; 38, 39).

## ‘Syngraft’ or ‘Isograft’ models retain species‐specific cross‐talk between tumour, stroma and immune cells

The two major limitations of all xenograft mouse models of human CRC cell lines are the species mismatch between tumour and stroma, which likely affects cell communication, and the use of immune‐deficient hosts. The role of the immune system appears to be particularly important for the development of CRC; T cell immune infiltrate is an important predictive criterion for patient survival 40 and mice with deletion of *Smad4* in T‐cells excessively secrete proinflammatory cytokines and develop gastrointestinal tumours 41. The use of immune‐deficient hosts allows development of tumours in the absence of an immune infiltrate, which is critical for CRC tumour progression, and also precludes the testing of immunomodulatory anti‐cancer agents. Grafting tumour fragments or the cell lines derived from them, for example MC38 cells (derived from a mouse colon adenocarcinoma), to a genetically identical inbred, immune competent mouse (‘syngraft’ or ‘isograft’) is a way of overcoming both of these problems 19.

## Genetically modified mouse models of colorectal cancer retain species‐specific cross‐talk between tumour, stroma and immune cells

Genetically engineered mouse models (GEMM) offer several advantages over cancer cell lines as research tools in cancer research. Despite their evolutionary distance, the gene content of the mouse and human genomes has been largely conserved through evolution 42, and most cancer pathways are operative in both species 43. The genomes of inbred laboratory mouse strains are well‐characterised and mice offer a ‘clean’ system in which to test the biological effects of genetic alterations or environmental effects; they are essentially homozygous at every locus and are housed in a controlled environment.

Sequencing of DNA from several tumour types that have developed in mouse or man has recently revealed that human tumours are more heterogeneous than their GEMM‐derived counterparts 44, 45. The difference in complexity of mouse and human tumours is likely due to a combination of factors. Humans have a more varied diet and a different microbiome compared to the laboratory mouse, and so their intestinal cells are exposed to more exogenous genotoxins 46. In addition, these gene‐environment interactions occur over a longer period of time in man; the development of tumours in GEMM is limited by the short lifespan of the mouse or by local research ethics; for example the UK Home Office enforces a tumour size limit of around 1 cm^2^. Furthermore, there is a lack of inter‐tumour heterogeneity of GEMM tumours because laboratory mice are inbred and therefore lack the genetic heterogeneity present in the outbred human population. Moreover, GEMM tumours are often initiated and maintained by the same genetic mutation of a strong cancer driver gene, which may limit the number of pathways to tumourigenesis. While the difference in complexity of tumours is advantageous when applying a cross‐species oncogenomics approach to cancer gene validation 45, the lack of complexity of mouse tumours poses a problem when trying to faithfully recapitulate the human disease for pre‐clinical studies. Some tumour types can be treated in mouse models of cancer, but it is thought that the heterogeneity of human tumours results in a greater potential for resistance to therapy to develop, or for recurrence after surgical resection. Nonetheless, GEMMs of CRC that develop autochthonous intestinal tumours can better model the dynamic interplay between intestinal tumour cells, stroma and the immune system, as well as the response to therapy, when compared with xenograft models.

## Genetically modified mouse models of late stage CRC—25 years in the making

Table 3 summarises some of the genetically modified mouse models (GEMM) of CRC that have been developed over the last 25 years. The first key mouse model of CRC was the multiple intestinal neoplasia (Min) mouse, which arose from a random ethylnitrosourea (ENU) mutagenesis screen 47, 48. The *Apc^Min/+^* mouse was subsequently recognised as a paralog for human familial adenomatous polyposis (FAP) syndrome and provided confirmation of the causal gene mutation in sporadic CRCs with 5q21 deletions 49; later studies showed that somatic inactivation of *APC* is observed in >80% of sporadic CRCs 2.

**Table 3:** Selection of genetically modified mouse models of colorectal cancer

Mouse allele	Rationale	Strategy	Advantages	Disadvantages
*Apc^Min/+^* 47, 48	• N/A	• Germline truncating mutation (N terminus)	• Model of human familial adenomatous polyposis (FAP) syndrome and >80% of sporadic CRCs contain mutations 2, 4	• Developed hundreds of low‐grade adenomas
	• Ethylnitrosurea (ENU) mutagenesis screen	• Loss of heterozygosity occurs at wildtype allele in tumours	• Short‐lived for rapid studies	• Short‐lived as tumour burden causes obstruction, prolapse and bleeding
			• Tumour multiplicity easily quantifiable	• Short life‐span means that adenomas do not acquire sufficient mutations to progress to adenocarcinoma and metastasise 56, 57
				• Tumours predominantly located in small rather than large intestine
*Msh2^−/−^* 54	*• Msh2* mutations common in CRC	• Germline knock‐out	• Model of Hereditary non‐polyposis CRC (HNPCC) or Lynch Syndrome (3% of all CRCs)	*• Msh2* mutation in all cells of body and mice are predisposed to lymphomas
*Villin‐Cre/Msh2^LoxP^* 55	• To restrict malignancy to intestine to prevent lymphoma	• Conditional	• Developed intestinal adenomas and adenocarcinomas	• Do not develop metastases
		*• Cre* recombinase^a^ expressed from promoter of intestinal specific gene (*Villin)*	• No deaths from lymphoma	
*Apc^580S/580S^* 64	• To model advanced CRC by restricting tumours to colon and reducing tumour burden	• Conditional Adenoviral Cre recombinase^a^ is administered through the anus to inactivate *Apc*	• Live >1 year.	• Do not develop metastases
			• Developed two or three intestinal adenomas	• Conditional allele (unactivated) reduces *Apc* expression in all cells, similar to *Apc^fl/fl^* 10, which results in development of life‐limiting hepatocellular carcinomas by 14 months (REM unpublished observations of *Apc^580S/580S^*)
			• Some mice developed adenocarcinomas	
*Apc^lox15/+^*; *Fabpl‐Cre* 109	• To model advanced CRC by restricting tumours to the colon and reducing tumour burden	• Conditional	• Live >1 year.	• Do not develop metastases
		*• Cre*‐recombinase^a^ is expressed in the distal small intestinal and colonic epithelia to inactivate *Apc*	• Developed two or three intestinal adenomas	
			• Some mice developed adenocarcinomas	
*Apc^Min/+^Trp53^−/−^* 110	• To model advanced CRC through addition of cooperating gene mutations	• Germline mutations in *Apc* and *Trp53*		• Do not develop metastases
*Apc^2lox14/+^ KrasLSL‐G12D* and *Fabpl‐Cre* 111	• To model advanced CRC through addition of cooperating gene mutations	• Conditional	• Developed more adenocarcinomas than single mutant (Apc or Kras alone) control mice	• Do not develop metastases
		*• Cre*‐recombinase^a^ is expressed in the distal small intestine/colonic epithelia and results in constitutive activation of K‐Ras and inactivation of Apc		
*Apc* mutant with disruption of *Tgfbr2, Smad2* or *Smad4*. 112, 113, 114	• To model advanced CRC through addition of cooperating gene mutations		• Developed more adenocarcinomas than single mutant control mice.	• Do not develop metastases
*Apc^Min/+^ Villin Cre Fbxw7^(ΔG)^* 115	• To model advanced CRC through addition of cooperating gene mutations	• Germline mutation of *Apc*	• Decreased lifespan	• Do not develop adenocarcinomas or metastases
		• Conditional mutation of Fbxw7	• Increased tumour burden	
		*• Cre* recombinase^a^ expressed from promoter of intestinal specific gene (*Villin)*	• Fbxw7 null control mice developed adenomas by 9‐10 months of age	
*Apc^CKO/CKO^‐LSL‐*G12D; *Kras^tm4tyj/+^* 62	• To model advanced CRC through addition of cooperating mutations. To restrict tumours to the colon and reduce tumour burden	• Adenoviral *Cre* solution administered via the anus to simultaneously disrupt *Apc* and activate K‐ras	• Developed adenocarcinomas after five months	• Labour intensive mouse surgery required to clamp a section of colon to deliver Adenoviral *Cre* solution to the colon via the anus
			• Developed metastases to distant organs after six months e.g. liver	
			• In vivo monitoring via colonoscopy	

CRC, Colorectal carcinoma.

^a^ *Cre* recombinases catalyse recombination between two *loxP* sites that are situated in the introns surrounding a critical exon(s) and result in excision of the DNA between the sites.

Hereditary non‐polyposis CRC (HNPCC) or Lynch Syndrome, is the most common inherited, autosomal dominant CRC syndrome and accounts for around 3% of all CRCs. HNPCC is most frequently caused by mutations in *MLH1, PMS1, PMS2, MSH2* or *MSH6*, (MutL Homolog 1, Post‐Meiotic Segregation Increased 1 and 2 and MutS Homolog 2 and 6, respectively) that encode the enzymatic machinery of DNA mismatch repair. As with sporadic cancers, intestinal cancers from HNPCC patients have a high frequency of mutations in *APC*, as well as *KRAS, TP53* and *TGFBR2* 50, 51, 52. Further, approximately 15% of sporadic CRCs display microsatellite instability (MSI) secondary to impaired DNA mismatch repair, frequently because of hypermethylation of the *MLH1* promoter 53. One of the earliest models that had the potential to recapitulate HNPCC was a homozygous *Msh2^−/−^* deficient mouse 54 (Table 3). Unfortunately, these mice were predisposed to lymphomas, which limited their use as a model of HNPCC. One way to restrict genetic modifications to tissues of interest is to use a *Cre*‐recombinase driven by a tissue‐specific promoter. Cre‐recombinases catalyse recombination between two *loxP* sites that are situated in the introns surrounding a critical exon(s) and result in excision of the DNA between the sites. Using a conditional strategy that results in expression of *Cre* recombinase from the *Villin* promoter (intestinal specific gene) a *Villin‐Cre/Msh2^LoxP^* mouse was generated that resulted in intestinal adenomas and adenocarcinomas in the absence of lymphoma 55 (Table 3).

The short lifespan of the *Apc^Min/+^* mouse limits the utility of the model and it was thought that mice with fewer tumours would live longer, and that if tumours were restricted to the colon, they might progress to carcinoma and metastasise 56, 57. A number of different *Apc* mutant mice with germline or inducible cell‐type specific conditional alleles of *Apc* were developed (see Table 3 for examples and for an extensive review, see 58). Despite evidence of malignant transformation and colonic invasion, aged *Apc* mutant mice did not develop metastases, suggesting that to better model the later stages of disease in the mouse, additional genetic mutations would be required (Table 3). *Apc* mutant mice have been crossed to mice with gene mutations found in later stage disease, including *TP53*, *KRAS*, *TGFBR2*, *FBXW7* and *SMAD2‐4*, in an attempt to generate more useful pre‐clinical models. At best, double mutants displayed more adenocarcinomas, but the mice did not model the metastatic disease (see Table 3).

The many different *Apc* mutant mice that have been created have led to greater understanding of the biology of Apc, for example, to ascertain which protein domains and functions of Apc are responsible for intestinal tumour initiation and maintenance; it is widely accepted that Apc's role in activation of the Wnt pathway is the major contributor to this process 59. *Apc* mutant mice have also been used to probe the effect of environmental factors and drugs on intestinal tumourigenesis and stem cell specific *Apc* deletion models lent support to the so‐called ‘bottom‐up’ model of adenoma formation 60, 61.

One of the most promising models for both the elucidation of the molecular events that support the formation of distant metastases of colorectal cancer and for preclinical investigations is a mouse that has simultaneous, inducible inactivation of *Apc* and activation of *Kras* in the adult colon (Table 3). Six months after surgery 20% of *Apc^CKO/CKO^‐LSL‐Kras* mice (G12D; *Kras^tm4tyj/+^* allele) mice had developed metastases to distant organs such as the liver 62. It is important to note that expression of wild‐type *Apc* may be disrupted by introduction of conditional alleles, which may elevate Wnt signalling levels in tissues other than the intestine, and which appears to drive the formation of life‐limiting hepatocellular tumourigenesis before the onset of intestinal tumourigenesis. For example *Apc* ^fl/fl^ and *Apc^580S/580S^* mice show reduced expression of Apc protein throughout their tissues (in the absence of Cre‐mediated recombination of alleles), and develop hepatocellular carcinomas between 9 and 15 months (our unpublished observations of *Apc^580S/580S^* mice; 63, 64). These findings may preclude the use of some mouse models in studies that aim to understand the events that drive the metastasis of CRC to the liver.

## Mouse models of inflammatory bowel disease and colorectal cancer

Inflammatory bowel diseases (IBD) such as ulcerative colitis predispose patients to developing colorectal cancer as a result of chronic inflammation. It is becoming apparent that these tumours may develop through alternative *TP53* dependent routes compared with classical *APC*‐driven tumours 65. In an attempt to model this disease process mice have been generated that are predisposed to intestinal specific inflammation, including *Il‐10^−/^* ^−^, *Il‐2^null^* and *Muc2^−/−^* deficient strains 66, 67. Colitis can also be experimentally induced by the administration of dextran sulfate sodium (DSS) to the drinking water of mice. In addition to mimicking IBD, mice treated this way also produce intestinal tumours 68. Azoxymethane (AOM) is another commonly used chemical that predisposes to cancer experimentally. AOM is thought to enable base mispairings, and when administered alone or in combination with DSS, AOM reliably produces tumourigenesis in wild type mice 69.

## Genetic screens in mice for validation of cancer ‘driver’ genes

**Figure 2:**
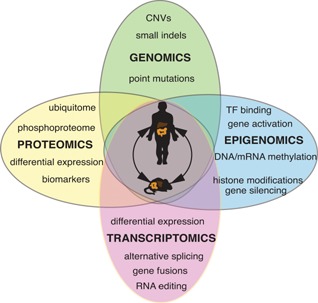
Cross‐species comparative ‘omics’ approaches for colorectal cancer (CRC) gene, therapeutic target and early disease biomarker discovery. Despite their evolutionary distance, the gene content of the mouse and human genomes has been largely conserved through evolution and most cancer pathways are operative in both species. Sporadic, low‐level mutagenesis of *Apc* and *Kras* in the mouse colon results in a high frequency of invasive adenocarcinomas and liver metastases. Furthermore, serial biopsies of patient tumours is not realistic, but with the development of mouse colonoscopes to guide biopsy collection, this is now a possibility in mouse models. Cancer gene, therapeutic target and biomarker discovery in patient specimens is challenging because of genetic, epigenetic and environmental heterogeneity between patients. Since many of these variables can be controlled for in the inbred laboratory mouse, the comparison of human and mouse primary and metastatic colorectal cancer genomes, transcriptomes, epigenomes, and proteomes including ubiquitinomes, will provide a powerful reductionist tool for identification of the molecular alterations that drive CRC invasion and metastasis. CNV, copy number variation; TF, transcription factor.

TCGA has identified genes that are frequently mutated in many different types of malignancy (frequently referred to as ‘pan‐cancer’ genes) suggesting that they are likely to accelerate the process 70. While these genes are obvious targets for therapy, many are considered intractable drug targets, including MYC and RAS. Within different cancer sub‐types there are cancer drivers that could be more amenable to therapy, however it is difficult to make the distinction between the ‘driver’ and ‘passenger’ genes (mutations that drive disease progression and mutations that do not) in patient specimens because of genetic, epigenetic and environmental heterogeneity. In contrast, mouse models, with their homogeneous genetic background and living environment, can be an efficient tool for validating candidate cancer genes (Figs. 1, 2). Forward genetic screens in mice using insertional mutagenesis have aided the identification or validation of many drivers of cancer, the relevance of which is assessed by comparing the candidates identified through screening with mutations found in the human cancer subtype 45.

One of the first insertional mutagenesis studies to identify novel genetic drivers of intestinal tumourigenesis involved restricting transposition to epithelial cells of the gastrointestinal tract using a *Villin‐Cre* recombinase system 71. Analysis of the common insertion sites for transposon in neoplasms, adenomas and adenocarcinomas revealed that 80% of insertions fell in known CRC genes including *APC*, *FBXW7 (*F‐box and WD repeat domain containing 7), *PTEN (*phosphatase and tensin homolog), and *SMAD4*, thus validating the approach. The remainder, including RSPO2 (R‐spondin 2), had not been previously implicated in the disease. R Spondin is a ligand for LGR4‐6 receptors (LGR, leucine‐rich repeat containing G protein‐coupled receptor), which stimulate the canonical Wnt signalling pathway. We now know that *RSPO2* fusion genes occur in a subset of colorectal carcinomas and are mutually exclusive with *APC* mutations 7.

To identify mutations that cooperate with inactivation of *Apc* and hyperactivation of the Wnt pathway, two independent teams of researchers mobilised the *Sleeping Beauty* transposon in *Apc^Min/+^* mice or conditional *Apc^fl/+^* mice 6, 72. The most frequently mutated gene (∼85%) in both studies was *Apc*, consistent with the requirement for loss of heterozygosity of *Apc* for intestinal tumour induction. Interestingly, all three mutagenesis screens identified a high number of insertions in the WW domain‐containing adaptor with coiled‐coil gene (*Wac*), which is also mutated in human cancers 6, 71, 72, 73. WAC has recently been shown to be required for activation of the cell‐cycle checkpoint in response to DNA damage suggesting that inactivation may cause genomic instability 74. Both insertional mutagenesis studies of *Apc* mutant mice identified common insertions in the voltage‐gated potassium channel subunit *Kcnq1* 6, 72. Mutations in voltage‐gated ion channel subunits have been identified in a variety of human cancers, including colon, and mounting evidence suggests that tumour cells have a depolarised membrane voltage relative to surrounding tissue, which could be exploited for therapy 75. In support of this finding, *Apc^Min/+^Kcnq1^−/−^* double mutant mice developed more intestinal adenomas than *Apc^Min/+^* control mice, and progression to aggressive adenocarcinomas was observed. The authors went on to show that reduced *KCNQ1* expression correlated with poor survival in patients; however, the exact mechanism of tumour suppression remains unknown 76. These examples prove the utility of transposon‐mediated insertional mutagenesis for rapid validation of colorectal cancer genes and for colorectal cancer gene discovery.

## *Apc^Min/+^* mice for early disease biomarker discovery

Most screening strategies aimed at the early detection of adenomas and CRC involve endoscopy, and thus have limited applicability. Early detection of CRC is critical for disease management, and so the identification of reliable, early disease biomarkers would improve the outcome for many patients. Mouse models that are predisposed to colorectal tumour formation can be an efficient tool for early disease biomarker discovery (Fig. 2). Since early detection of CRC is critical for disease management, investigators have used mice with germline *Apc* mutations, which model the initiation of intestinal tumourigenesis, to screen for early disease biomarkers. Clusterin was a ‘proof‐of‐principle’ for the cross‐species approach to biomarker discovery 77. Upregulation of clusterin was reported in adenomas from *Apc^Min/+^* mice and human tumours, and was recently shown to be highly‐expressed in intestinal stem cells 77, 78. Secreted clusterin is cytoprotective and its prosurvival function forms the basis of the current Phase I/II clinical trials against prostate, lung, and breast cancers 79.

### Cross‐species proteomics identifies early disease biomarkers

Recent improvements in proteomic profiling methods, based primarily on mass spectrometry, have allowed the detection and identification of peptides in the blood and biological fluids of cancer patients (Fig. 2). The plasma of tumour‐bearing *Apc^580S/^^Δ^* mice was screened by mass spectrometry, and cathepsin B and D were found to be elevated compared with control mice 80. Cathepsins are involved in the degradation of the basement membrane and extracellular matrix, a key step in tumour invasion and metastasis. Cathepsin B and cathepsin D are thought to associate with tumour grade of CRC and survival suggesting they may be useful biomarkers of the disease 80, 81. Similarly, proximal fluid proteome profiling of colon tumours from aged *Apc^15lox/+^ Fabpl‐Cre* mice resulted in the identification of 192 proteins that were more highly excreted by tumours relative to control, including Chitinase 3‐like 1 (*Chi3l1*) 82. CHI3L1 is thought to be involved in processes such as inflammation and tissue remodeling, and was shown to be significantly elevated in the sera of patients with adenomas and advanced adenomas compared with control individuals 83.

### Cross‐species epigenomics identifies epigenetic signatures of early disease

Epigenomic profiling of adenomas from *Apc^Min/+^* mice has also been useful in the identification of patient relevant, early disease biomarkers (Fig. 2). Immunoprecipitation of methylated DNA followed by sequencing (known as ‘MeDIP‐seq’) identified regions of the genome were differentially methylated 84. These regions were mainly enriched for targets of the Polycomb Repressive Complex, the subunits of which, together with those for several DNA methyltransferase complexes, were up‐regulated in adenomas. This genome‐wide pattern was shown to be partly conserved in human colon carcinomas, suggesting that an epigenetic signature is established early and is retained during progression from adenoma to carcinoma.

## Target identification and validation requires cancer cell lines, xenograft models and GEMM

Not all cancer genes are essential for the sustained growth of established tumours. In addition, tumours may have intrinsic therapeutic resistance, which is believed to arise through natural selection of a latent subclone within a tumour, or acquired therapeutic resistance, which arises through acquisition of a genetic mutation that allows the cancer cell to survive in the presence of the therapeutic agent 15. It is therefore important to understand both the contribution of a disease target to tumourigenesis and the mechanisms by which cancers evade targeted therapies. Here, we highlight several recent approaches to the identification and validation of novel therapeutic targets using mouse models of CRC.

Although genetic ablation or overexpression of potential therapeutic targets in CRC cell lines before xenotransplantation does not necessarily mimic pharmacological intervention, nor does it mimic the biology of autochthonous tumours, it may provide a shortcut to target validation (Fig. 1). This approach has recently been useful for the validation of potential therapeutic targets within the Wnt and TGFβ pathways.

### Targeting the Wnt pathway—reality or drug discovery pipe dream?

Since the Wnt–β‐catenin signalling pathway is hyperactivated in 93% of colorectal tumours and high Wnt activity defines colon cancer stem cells, the Wnt pathway is the most attractive therapeutic target for colorectal cancer 2, 29, 85. Despite this, therapeutic agents that specifically target the Wnt pathway have only recently entered clinical trials, and none has yet been approved. The Wnt inhibitor XAV939 inhibits the poly(ADP)‐ribosylating (PARP) enzymes tankyrase 1 and tankyrase 2, which interacts with AXIN (acts with APC to destruct β‐catenin and damper Wnt pathway activity) to promote its ubiquitylation and degradation 86. The development of more selective and potent second‐generation tankyrase inhibitors is in progress, however their anti‐tumour potential may not be fully realised owing to their intestinal toxicity in pre‐clinical mouse models 9. Rosenbluh and colleagues took a more targeted approach to inhibition of the Wnt pathway: they screened 85 cancer cell lines and found that β‐catenin active cancers are dependent on a signalling pathway involving the transcriptional regulator YAP1 (Yes‐associated protein 1) 28. YAP1 and the transcription factor TBX5 were shown to form a complex with β‐catenin, and phosphorylation of YAP1 by the tyrosine kinase YES1 lead to localisation of the complex to the promoters of anti‐apoptotic genes such as *BIRC5*. To determine whether YAP1 was required for HCT116 xenograft growth they transfected cells with a doxycycline‐inducible shRNA to YAP1. Three weeks after injection, tumour growth was restricted to 80% of control animals, suggesting that the YAP1 pathway might be an attractive therapeutic target 28. Further studies will be required to confirm whether this effect occurs in vivo, where many endogenous ligands that are secreted by stromal cells are known to modify Wnt pathway activity 22.

### Targeting MYC—drugging the undruggable

The transcription factor Myc proto‐oncogene protein (Myc) is thought to be a target of the β‐catenin–Tcf4 transcription complex in CRC cells in vitro, in normal crypts in vivo and in intestinal epithelial cells acutely transformed on in vivo deletion of the *Apc* gene 59, 87, 88. Array expression analysis revealed that Myc is required for the majority of β‐catenin–Tcf4 transcription complex gene activation following *Apc* loss in mice. While Myc is required for the formation of intestinal crypts, it is dispensable for homeostasis of the adult intestinal epithelium 89. Using a combination of conditional mouse models, it was shown that simultaneous inactivation of *Myc* and *Apc* completely ablated tumour development in mice despite high levels of nuclear β‐catenin in the intestinal epithelium 90. Multiple cancer‐associated single‐nucleotide polymorphisms (SNPs) have been mapped to conserved sequences within a 500‐kilobase region upstream of the *MYC* oncogene on human chromosome 8q24, and mice lacking a *Myc* enhancer (Myc‐335) that includes human SNP rs6983267 are resistant to intestinal tumours induced by the *ApcMin* mutation 91. While these studies have established Myc as a critical mediator of the early stages of intestinal neoplasia following *Apc* loss, it has not yet been possible to pharmacologically regulate Myc. Instead, a selective small‐molecule BET bromodomain inhibitor, JQ1, inhibits proteins that are regulatory factors for Myc 92. Treatment with JQ1 resulted in genome‐wide downregulation of Myc‐dependent target genes, reduced proliferation and had anti‐cancer properties in a mouse model of multiple myeloma, a Myc‐dependent hematologic malignancy, suggesting that JQ1 may perform similarly in mouse models of colorectal cancer.

### Pharmacological inhibition of TGFBR1 inhibits metastasis formation

A large proportion of CRCs display mutational inactivation of the TGFβ pathway. However, paradoxically, they are characterised by elevated TGFβ production. In other cancer types, such as breast or prostate, which retain a functional TGFβ signalling pathway, TGFβ induces a variety of prometastatic programmes that range from induction of epithelial‐to‐mesenchymal‐like transition (or ‘EMT’) to expression of genes that allow colonisation of foreign organs 93. During 10 years of follow‐up after surgical resection of colorectal tumours, only patients with medium or high TGFβ expression in the primary tumour suffered cancer recurrence, whereas all patients bearing TGFβ ‐low stage I, II and III tumours remained disease‐free 94. When Nude mice were inoculated with KM12L4a cells that overexpressed TGFβ, nearly all developed lung and/or liver metastasis, whereas only one third of control mice developed metastases. It is thought that the metastatic KM12L4a^TGFB1^ cells were conferred a survival advantage by suppressing apoptotic stimuli encountered during organ colonization. Moreover, inhibition of TGFBR1 has been shown to inhibit metastasis formation in multiple xenograft models 94, 95. Since the mechanism is thought to involve cross‐talk between tumour and stromal cells, it will be important to show that this regulation occurs in autochthonously occurring liver metastases of CRC, such as those that arise in the conditional *Apc^CKO/CKO^/Kras^G12D^* colon cancer model 62.

### Isograft models for validating the role of the immune system in CRC

In a recent study, Bindea and colleagues examined the spatio‐temporal dynamics of 28 different immune cell types (the ‘immunome’) that were infiltrating colorectal tumours were analysed by whole‐genome expression, PCR arrays and multicolored immunohistochemistry 96. The analysis revealed that patients with elevations in genes related to MHC‐II, B cell co‐stimulation, T cells and Tfh cells experience increased survival. One chemokine in particular, CXCL13, and one cytokine, IL‐21, positively correlated with disease‐free survival. Bindea and colleagues showed growth acceleration of MC38 isografts in *Cxcr5^−/^* ^−^ mice (CXCR5 is the receptor for CXCL13) compared to control, and rejection of tumour cells when recombinant CXCL13 was injected into the colonic submucosa of wild‐type mice before transplantation 96. These data confirmed the prognostic value of CXCL13 in assessing CRC tumour burden, and prove the utility of mouse models in the validation of disease biomarkers.

## Conclusions and outlook

In combination with studies of CRC cell lines and organoid models, mouse models of late‐stage CRC will help us to build a comprehensive picture of the molecular alterations that drive tumour formation and disease progression, through cross‐species analysis of the genomes, epigenomes, transcriptomes, proteomes and even immunomes of cancers of mice and men. Mouse models will be critical for reductionist approaches that aim to validate the cancer drivers from mutations identified by the TCGA.

Human CRC is the result of interactions between genes, including those involved in host immunity, and the environment, including diet and the microbiome 97, 98. The mouse affords the ability to investigate the effects of each of these factors independently of each other, which is simply not possible in man. However, recent studies have shown that ‘pound for pound’, the average mouse tumour is less complex than the average human tumour. These sequencing studies, together with the knowledge that lifestyle factors strongly influence the chances of getting cancer, suggest that the relative simplicity of GEMM tumours is due to the lack of gene‐environment interactions in the controlled environment of the laboratory mouse. There is a reasonable body of evidence to suggest that high red meat intake and alcohol consumption are risk factors for CRC, but we are probably only just beginning to understand how our environment affects the course of disease 99. Modelling the full heterogeneity of human tumours will be required to help tackle the problems of therapeutic resistance and recurrence after surgical resection in CRC; this might be better achieved by generating GEMM that combine a genetic predisposition to CRC with a diet rich in foods associated with increased risk of CRC.
